# *Entamoeba histolytica* in Different Water Sources of Niğde Province of Turkey

**DOI:** 10.1007/s11686-024-00886-z

**Published:** 2024-08-20

**Authors:** Cemal Candan, Mustafa Karatepe, Bilge Karatepe

**Affiliations:** 1 Graduate School of Natural and Applied Sciences, Department of Biotechnology , Niğde Ömer Halisdemir University, Niğde, Türkiye; 2 Faculty of Science, Department of Biotechnology, Niğde Ömer Halisdemir University, Niğde, Türkiye

**Keywords:** ELISA, *Entamoeba histolytica*, Niğde, Turkey, Water

## Abstract

**Purpose:**

This study was carried out to determine the presence of *Entamoeba histolytica* in water sources of Niğde province in Turkey, between June and November 2021.

**Methods:**

A total of 90 water samples were taken from 15 different water sources (drinking water, well water, spring water, wastewater and dam water) every month and the presence of *E. histolytica* antigens in the samples was examined by ELISA.

**Results:**

The positivity for *E. histolytica* was determined in 7 (7.7%) of 90 samples. While no antigens were found in any of the samples in June and September, *E. histolytica* was positive for three samples (20%) in July, one sample (6.6%) in August and October and two samples in November (13.3%). One of 24 dam samples (4.1%), 1 of 12 wastewater samples (8.3%), 1 of 12 well samples (8.3%), and 4 of 24 fountain samples (16.6%) that examined by ELISA were found positive. On the other hand, none of the examined 18 spring samples were positive. In addition, 4 (8.8%) of 45 samples that examined in summer and 3 (6.6%) of 45 samples that examined in autumn were detected positive by using ELISA. *Entamoeba histolytica* positivity in samples was statistically insignificant in terms of months, water resources and seasons (*P* > 0.05).

**Conclusion:**

As a result, the presence of *E. histolytica*, which is an important public health problem in water sources, was determined for the first time in Niğde province of Türkiye with this study.

## Introduction

Amebiasis is one of the protozoan diseases in the intestines caused by *E. histolytica*. *Entamoeba histolytica* is more prevalent in developing countries and is generally transmitted to humans through cysts found in feces or contaminated food and water [1]. It is estimated that approximately 10% of the world’s population is infected with this parasite, with distribution in Turkey ranging from 0 to 17% [2, 3]. While *E. histolytica* primarily infects humans, it can also parasitize various monkey species, dogs, cats, pigs, cattle, and rats. However, it is noted that the parasite does not form cysts in hosts other than humans. The parasite usually settles in the intestines of hosts but can also migrate to other organs such as the liver, lungs, brain, and spleen, causing abscesses [3–5].

*Entamoeba histolytica* generally causes asymptomatic infections, but in some cases, it can lead to symptoms ranging from a few watery stools to profuse bloody diarrhea. Symptoms may include foul-smelling stools, weakness, and fatigue [3, 6, 7].

The diagnosis of amebiasis is made through various laboratory analyses. The diagnosis of protozoan infections can be established through symptoms, direct microscopic examination, and methods such as ELISA and PCR. Serological tests are crucial for diagnosing amoebiasis, as the diagnostic level of the parasite remains very low, even when at least three different stool samples are microscopically examined [6, 7].

In this study, the aim was to determine the presence of *E. histolytica*, which is important for human health, in water samples taken from different water sources in the Niğde province of Turkey by ELISA.

## Materials and Methods

### Study Area

Niğde is located at an altitude of 1,299 m in the Central Anatolia Region of Türkiye. According to the 2020 statistics, the population of Niğde city center is 230.776 people. Niğde experiences the typical continental climate of Central Anatolia, with hot and dry summers and cold and snowy winters. Rainfall is generally in the form of snow in winter and rain in the spring. The average temperatures indicate that July is the hottest month, while January is the coldest. Showers are observed in the form of heavy rain in April, May, and the early days of June. The average precipitation in the city is 0.9 mm. April has the highest rainfall with 78.5 mm, while July has the lowest with 0.2 mm.

### Field Study

Water samples were collected from 15 different water sources every month, between June 2021 and November 2021 in the Niğde province. The samples were collected in 5 L clean plastic containers. The collected water samples included 4 from dam waters used for agricultural irrigation, 2 each from wastewater and well water, 3 from spring water, and 4 from fountains used for drinking water purposes.

### Laboratory Study

The sample number was chosen considering these many samples were proven to represent the population adequately in previous studies [8, 9]. A total of 90 water samples were stored at 4 °C until they were processed in the laboratory. A vacuum pump filtration device with a 0.45 μm cellulose acetate membrane filter (Ø, 45 mm) was used for filtering the water samples. Subsequently, the filter was transferred to a clean tube, and 20 ml of the same water sample was added. After vortexing to remove particulate matter, the sample was centrifuged at 2500 rpm for 10 min to discard the upper portion, and the 1.5 ml sediment at the bottom was transferred to an eppendorf tube and stored in a deep freezer at -20℃ until further analysis with the ELISA.

### ELISA (Enzyme Linked Immunosorbent Assay)

A commercial ELISA kit (*E. histolytica* II™, TECHLAB 2001 Kraft Drive Blacksburg, VA 24060 − 6358 USA) was used to investigate the specific adhesin antigen of *E. histolytica* in samples collected from different water sources. The ELISA was performed following the procedure provided by the manufacturer.

ELISA results were read at 450 nm wavelength using an ELISA microplate reader, MR-96 A, and the obtained values were calculated using the kit procedure.

### Statistical Analysis

In the conducted study, the chi-square test was used for the statistical evaluation of positivity values concerning the months of water sample collection, water sources, and seasons. Therefore, the SPSS (Statistical Programme for Social Science) for Windows 24.0 Statistical Package Program was utilized.

## Results

Out of the total 90 water samples collected from 15 different water sources in the Niğde province, the presence of *E. histolytica* was detected in seven samples (7.7%) through the ELISA.

**Table 1:** *Entamoeba histolytica* presence in water sources with regard to months

Months	Number of Examined Sources	Number of Positive Samples	Positives (%)
June	15	-	-
July	15	3	20
August	15	1	6.6
September	15	-	-
October	15	1	6.6
November	15	2	13.3
**Total 90**		**7**	**7.7**

In Table 1, the presence of *E. histolytica* is given according to the months. Accordingly, for water samples taken from 15 different sources, in June and September, none of them showed *E. histolytica* presence. In July, three samples (Uluagac, Yesilburc, Okcu) showed positivity at a rate of 20%. In August (Kemerhisar) and October (Alaaddin), one sample each showed positivity at a rate of 6.6%. In November, two samples (Alaaddin, Kizilca Creek) showed positivity at a rate of 13.3%. *Entamoeba histolytica* positivity of water samples by months was found to be statistically insignificant (*p* > 0.05).

**Table 2:** *Entamoeba histolytica* presence according to the sampled water sources

Sampled Water Sources	Study Center	Number of Examined Samples	Number of Positive Samples	Positives (%)
Dam	Gumusler, Akkaya, Uluagac*, Gebere	24	1	4.1
Wastewater	Kizilca Creek*, Yesil Creek	12	1	8.3
Well Water	Kemerhisar*, Tepe Garden	12	1	8.3
Spring Water	Kumluca, Hancerli, Roma Pool	18	0	-
Fountain	Yesilburc*, Alaaddin**, Sadirvan, Okcu*	24	4	16.6
**Total**	**90**		**7**	**7.7**

*: Positive samples

In Table 2, E. *histolytica* presence is presented according to the water sources from which the samples were collected. Accordingly, out of the 24 dam water samples examined with the ELISA test, 1 (4.1%) was found to be positive. Among the 12 wastewater samples, 1 (8.3%) was positive, as well as 1 (8.3%) out of the 12 well water samples. Additionally, 4 (16.6%) out of the 24 fountain water samples were found to be positive. In contrast, none of the 18 spring water samples examined showed the presence of *E. histolytica*. When the collected water samples were examined in terms of *E. histolytica* positivity rates based on water sources, it was determined to be statistically insignificant (*p* > 0.05).

**Table 3:** *Entamoeba histolytica* presence according to seasons

Seasons	Study Center	Number of Examined Samples	Number of Positive Samples	Positives (%)
Summer	Uluagac*, Yesilburc*, Okcu*, Kemerhisar*	45	4	8.8
Autumn	Alaaddin**, Kizilca Creek*	45	3	6.6
**Total**	**90**		**7**	**7.7**

*: Positive samples

In Table 3, E. *histolytica* presence determined according to seasons for the collected water samples is provided. Accordingly, out of the 45 water samples examined with the ELISA test during the summer months, 4 (8.8%) were positive, and out of the 45 samples examined in the fall months, 3 (6.6%) were positive. The presence of *E. histolytica* was detected in water samples during the summer and fall months, and the positivity determined in terms of seasons was found to be statistically insignificant (*p* > 0.05).

## Discussion and Conclusion

Amebiasis caused by *E. histolytica* is widespread in Turkey and worlwide. Amoebic dysentery is frequently occured in developing and underdeveloped countries. *Entamoeba histolytica* exists in our environment as a cyst form and is transmitted to humans orally by inadequately sterilized vegetables, fruits, and drinking water [3, 10].

In many countries around the world, various studies have been conducted on waterborne protozoa in different water sources, and mostly *E. histolytica*, *G. intestinalis* (*G. lamblia*), and *C. parvum* species have been identified [11–23]. In this study, *E. histolytica* was found positive in neighbourhood fountains, well water, wastewater, and dam waters examined by ELISA. In contrast, no parasites were found in any of the 18 spring water samples examined similar to elsewhere [11–23]. In this context, the detected *E. histolytica* positivity in different water sources suggests fecal contamination, which should be used after effective treatment and disinfection.

In Turkey, the presence of protozoan parasites in different water sources revealed protozoa such as *G. intestinalis*, *C. parvum* and *T. gondii* that have been investigated more than *E. histolytica* [24–31]. In a study conducted in Ankara, samples taken from various water sources were examined for standard microscopic, IFA, ELISA, and PCR techniques. Two of the examined well water samples were found to have *G. intestinalis*, two of the river water samples had *E. histolytica*, and one had *C. parvum*, no parasites were detected in municipal waters and dam water [8]. In this study, however, it was found that 7 out of a total of 90 water samples taken from 15 different water sources in Niğde province were positive for *E. histolytica* by ELISA test, and the prevalence of *E. histolytica* was determined to be 7.7%. Of the positive samples, 4 were neighbourhood fountains (Yesilburc, Okcu, and Alaaddin), 1 was a dam (Uluagac), 1 was well water (Kemerhisar), and 1 was wastewater (Kizilca Creek). It was observed that the results obtained from various water sources for *E. histolytica* in this study conducted in the Niğde province differed from the results in Ankara. The difference may be due to the contamination levels of the water samples, the disinfection methods applied, and the detection methods used.

Amebiasis caused by *E. histolytica* is widespread worldwide, it is more common in tropical and subtropical regions. In Niğde region, the presence of *E. histolytica* in humans was found microscopically in fecal samples of university students at a rate of 2.12% [32], and in primary school students at a rate of 13.34% [33]. This situation suggests that people in the Niğde province are infected with this parasite (2.12–13.34%), and when there is a problem in the sewage systems, water sources may become contaminated fecally. Thus, waters containing *E. histolytica* may pose a risk to public health.

In conclusion, the presence of *E. histolytica* in different water sources (drinking water, well water, spring water, wastewater, and dam water) in the Niğde province is reported for the first time in this study. The presence of *E. histolytica* in 7.7% of water sources was determined by ELISA. ELISA is among the most popular methods used in diagnostic laboratories throughout the world. When the test protocol is applied correctly, ELISA kits have very high sensitivity and specificity (> 95%). The *E. histolytica*-specific ELISA offers the significant advantage of the rapid differentiation of *E. histolytica* from *E. dispar* [34, 35]. In addition, further investigations, including molecular techniques are required in order to determine the importance of this parasite as a cause of clinical disease in human in Niğde province. However, it is less expensive and relatively easier to detect the protozoan species with ELISA compared to PCR analysis. This study emphasize the importance of an adequately and appropriately disinfection treatment of drinking water, well water, wastewater, and dam waters in terms of public health in the Niğde province. It is crucial to increase public awareness of the impact of *E. histolytica* on public health in the Niğde province to contribute to the prevention of waterborne parasitic epidemic diseases and ensure people’s access to safe and healthy water.

## References

[1] Current W, Garcia L (1991) Cryptosporidiosis. Clin Microbiol Reviews 4:325–358. 10.1128/cmr.4.3.325 10.1128/cmr.4.3.325 PMC358202 1889046

[2] Özçelik S, Malatyalı E (2008) Amebiasis and the role of Virulance factors in *Entamoeba histolytica* Pathogenesis. Türk Hijyen Ve Deneysel Biyoloji Dergisi 65:139–148

[3] Yukarı BA (2013) Entamoebiasis. In: Özcel MA, Yukarı BA (eds) Veteriner Hekimliğinde Parazit Hastalıkları. Meta Basım, İzmir

[4] Soulsby EJL (1986) Helminths, Arthropods and Protozoa of Domesticated animals. Bailliere and Tindall, London

[5] Taylor MA, Coop RL, Wall RL (2016) Veterinary parasitology. Blackwell Publishing, Oxford, UK

[6] Doganci L, Tanyuksel M, Doganci T (2004) Accurate diagnosis is essential for amebiasis. World J Gastroenterol 10:1231. 10.3748/wjg.v10.i8.1231 15069733 10.3748/wjg.v10.i8.1231 PMC4656368

[7] Haque R, Huston CD, Hughes M, Houpt E, Petri WA (2003) Entamoeba histolytica. N Engl J Med 348:1565–1573. 10.1056/NEJMra022710 12700377 10.1056/NEJMra022710

[8] Bakir B, Tanyuksel M, Saylam F, Tanriverdi S, Araz RE, Hacim AK, Hasde M (2003) Investigation of waterborne parasites in drinking water sources of Ankara, Turkey. J Microbiol 41:148–151

[9] Koloren Z, Sotiriadou I, Karanis P (2011) Investigations and comparative detection of *Cryptosporidium* species by microscopy, nested PCR and LAMP in water supplies of Ordu, Middle Black Sea, Turkey. Annals Trop Med Parasitol 105:607–615. 10.1179/2047773211Y.0000000011 10.1179/2047773211Y.0000000011 PMC4089801 22325820

[10] Yukarı BA (2015) Entamoebidae, Hartmannellidae, Vahlkampfidae. In: Dumanlı N, Karaer KZ (eds) Veteriner Protozooloji. Medisan Yayınevi, Ankara

[11] Ferrer A, Nguyen-Viet H, Zinsstag J (2012) Quantification of diarrhea risk related to wastewater contact in Thailand. EcoHealth 9:49–59. 10.1007/s10393-012-0746-x 22311100 10.1007/s10393-012-0746-x

[12] Akbar N, Ayaz S, Rahman S, Khan S, Khan SN, Noor AA, Shagufta BI, Raza F, Waqar M (2014) Molecular detection of *Entamoeba histolytica* in different water sources of District Peshawar, Pakistan. Annual Res Rev Biology 1:1461–1470. 10.9734/ARRB/2014/7935 10.9734/ARRB/2014/7935

[13] Ghasemi E, Rahdar M, Rostami M (2015) Prevalence of *Entamoeba histolytica*/*dispar* in drinking water in the city of Shush, Khuzestan Province in 2011. Int J Curr Microbiol Appl Sci 4:582–588

[14] Mahmoudi MR, Nazemalhosseini-Mojarad E, Karanis P (2015) Genotyping of *Giardia lamblia* and *Entamoeba* spp from river waters in Iran. Parasitol Res 114:4565–4570. 10.1007/s00436-015-4702-x 26350378 10.1007/s00436-015-4702-x

[15] Hemmati A, Hooshmand E, Hosseini MJ (2015) Identification of *Entamoeba histolytica* by molecular method in surface water of Rasht city, Iran. Iran J Public Health 44:238–243 25905058 PMC4401882

[16] Berglund B, Dienus O, Sokolova E, Berglind E, Matussek A, Pettersson T, Lindgren PE (2017) Occurrence and removal efficiency of parasitic protozoa in Swedish wastewater treatment plants. Sci Total Environ 598:821–827. 10.1016/j.scitotenv.2017.04.015 28458199 10.1016/j.scitotenv.2017.04.015

[17] Ajonina C, Buzie C, Möller J, Otterpohl R (2018) The detection of *Entamoeba histolytica* and *Toxoplasma Gondii* in wastewater. J Toxicol Environ Health 81:1–5. 10.1080/15287394.2017.1392399 10.1080/15287394.2017.1392399 29173133

[18] Elfadaly HA, Hassanain NA, Hassanain MA, Barakat AM, Shaapan RM (2018) Evaluation of primitive ground water supplies as a risk factor for the development of major waterborne zoonosis in Egyptian children living in rural areas. J Infect Public Health 11:203–208. 10.1016/j.jiph.2017.07.025 28843417 10.1016/j.jiph.2017.07.025

[19] Sabbahi S, Trad M, Ben Ayed L, Marzougui N (2018) Occurrence of intestinal parasites in sewage samples and efficiency of wastewater treatment systems in Tunisia. Water Qual Res J 53:86–101. 10.2166/wqrj.2018.033 10.2166/wqrj.2018.033

[20] Al-Khalidy KAH, Abbas AJ (2020) Detection of some intestinal parasites in the wastewater of some health centers (popular clinics) in some areas of Al-Diwaniyah city, Iraq. Eurasian J Biosci 14:2115–2121

[21] Al-Nihmi FM, Salih AA, Qazzan J, Radman B, Al-Woree W, Belal S, Al-Motee J, Ahlam AA, Al-Harthee A, Al-Samawee H, Al-Samawee B, Atiah H (2020) Detection of pathogenic waterborne parasites in treated wastewater of Rada’a City-Yemen. J Sci Res Med Biol Sci 1:30–39. 10.47631/jsrmbs.v1i1.23 10.47631/jsrmbs.v1i1.23

[22] Shankar P, Mishra J, Bharti V, Parashar D, Singh S (2019) Multiplex PCR assay for simultaneous detection and differentiation of Entamoeba histolytica, Giardia lamblia, and Salmonella spp. in the municipality-supplied drinking water. J Lab Physicians 11:275–280. 10.4103/JLP.JLP_66_18 31579243 10.4103/JLP.JLP_66_18 PMC6771313

[23] Danjuma FY, Onaji AI, Ocheme JO, Danladi YP (2020) Analysis of physicohemical parameters and prevalence of *Entamoeba histolytica* in water drawn from cast and non-cast wells in Zaria, Nigeria. Int J Adv Res 8:1298–1305. 10.21474/IJAR01/11046 10.21474/IJAR01/11046

[24] Demirel E (2014) Detection of *Toxoplasma gondii* in the Water Samples of Ordu and Giresun Provinces by Molecular Techniques. Dissertation, University of Ordu

[25] Ayaz E (2015) Investigation on *Cryptosporidium parvum* by using Molecular Technics in Water Samples Collected From Provinces of Giresun and Samsun. Dissertation, University of Ordu

[26] Kaya Y, Karaman Ü, Yolalan G (2021) Evaluation of Spring Waters in Ordu Province in terms of Parasite Presence. ODÜ Tıp Dergisi 8:14–18

[27] Koloren Z, Demirel E (2013) Detection of *Toxoplasma Gondii* in Turkish river and drinking water samples by different PCR and LAMP methods. CLEAN–Soil Air Water 41:963–968. 10.1002/clen.201200468 10.1002/clen.201200468

[28] Kolören Z, Karaman Ü (2017) Determination of waterborne parasites in environmental water samples collected from Terme and Kocaman Rivers in Samsun province of the Black Sea Region. Akademik Ziraat Dergisi 6:177–182. 10.29278/azd.371077 10.29278/azd.371077

[29] Sağlam T (2018) A Research on Some Single Cells (Protozoa) Parasites in Agricultural Irrigation and Drinking Water Resources in Denizli City Center. Dissertation, University of Pamukkale

[30] Sağlam T, Düşen S, Yağcı MA, Yağcı A (2021) Investigation of *Cryptosporidium* spp. and *Giardia* spp. in Lake Eğirdir (Isparta). Türk Mikrobiyoloji Cemiyeti Dergisi 51:363–367. 10.5222/TMCD.2021.87004 10.5222/TMCD.2021.87004

[31] Seferoğlu O (2014) Detection of *Giardia intestinalis* in the Water Samples of Samsun and Giresun Provinces by Molecular Techniques, Dissertation, University of Ordu

[32] Sarıaslan Y, Doğanay M, Türkmen H (2001) The prevalence of intestinal parasites in female students between 17 and 25 years of age in the Sabancı dormitory in Niğde, Turkey. Türkiye Parazitoloji Dergisi 25:66–68

[33] Koz TB (2003) The prevalence of the intestinal and ectoparasites among the primary school students in the center of Niğde and its villages. Dissertation, University of Niğde

[34] Tanyuksel M, Petri WA (2003) Laboratory diagnosis of Amebiasis. Clin Microbiol Rev 16:713–729. 10.1128/cmr.16.4.713-729.2003 14557296 10.1128/cmr.16.4.713-729.2003 PMC207118

[35] Fotedar R, Stark D, Beebe N, Marriott D, Ellis J, Harkness J (2007) Laboratory Diagnostic techniques for *Entamoeba* Species. Clin Microbiol Rev 20:511–532. 10.1128/cmr.00004-07 17630338 10.1128/cmr.00004-07 PMC1932757

